# Supplementary material for: The Application and Challenges of Brain Organoids in Exploring the Mechanism of Arbovirus Infection
Source: Microorganisms. 2025 May 30;13(6):1281. doi: 10.3390/microorganisms13061281 (PMC12195329; doi:10.3390/microorganisms13061281)
Supplement: Supplementary file 1 [file microorganisms-13-01281-s001.zip › Table S1 Comparative analysis of brain organoid models for arbovirus research.pdf]

Table S1 Comparative analysis of brain organoid models for arbovirus research

Origin	Types of brain organoid	Virus	Number of applications	Key differentiation factors	Culture method
hESC	Brain organoids (Cerebral organoids)	ZIKV	6	N2/B27 supplementation, SMAD inhibitor induces neural differentiation, bFGF/EGF promotes proliferation, BDNF/GDNF facilitates maturation, Matrigel provides 3D support	1.Stir flask bioreactors<br>2.rotary culture<br>3.oscillatory culture
hESC	Cortical organoids	ZIKV,JEV	2	IWR-1/SB-431542 induces dorsal telencephalic differentiation, FGF/EGF promotes neurosphere proliferation, and BDNF/GDNF/IGF-1/NT3 drives neuronal maturation	/
hiPSC	Forebrain-specific organoids	ZIKV	2	ROCK inhibitors form cell aggregates, neural differentiation is initiated using SMAD inhibitors, WNT modulators (IWR/XAV), N2/B27 maintains growth, and neuronal maturation is promoted by BDNF/GDNF.	1.rotary culture
hiPSC	Brain organoids (Cerel organoids)	ZIKV,LACV	7	N2/B27, SMAD inhibitor initiates neural differentiation, bFGF/EGF promotes neural precursor amplification, Matrigel provides 3D support, and neurotrophic factors such as BDNF/GDNF promote maturation	1.oscillatory culture<br>2.rotary culture<br>3.Stir flask bioreactors

Note：/: Not available
